# Efficient ReSe_2_ Photodetectors with CVD Single-Crystal Graphene Contacts

**DOI:** 10.3390/nano11071650

**Published:** 2021-06-23

**Authors:** Bruna Silva, João Rodrigues, Balaji Sompalle, Chun-Da Liao, Nicoleta Nicoara, Jérôme Borme, Fátima Cerqueira, Marcel Claro, Sascha Sadewasser, Pedro Alpuim, Andrea Capasso

**Affiliations:** 1 International Iberian Nanotechnology Laboratory, 4715-330 Braga, Portugal; bruna.silva@inl.int (B.S.); joao.rodrigues@inl.int (J.R.); sgds.balaji@gmail.com (B.S.); chundaliao@ntu.edu.tw (C.-D.L.); nicoleta.nicoara@inl.int (N.N.); jerome.borme@inl.int (J.B.); fatima.cerqueira@inl.int (F.C.); marcel.claro@inl.int (M.C.); sascha.sadewasser@inl.int (S.S.); pedro.alpuim.us@inl.int (P.A.); 2 Centre of Physics, University of Minho, 4710-057 Braga, Portugal

**Keywords:** 2D materials, transition metal dichalcogenides, van der Waals heterostructures, hexagonal boron nitride, CVD, optoelectronics, contact barrier height

## Abstract

Rhenium-based 2D transition metal dichalcogenides such as ReSe_2_ are suitable candidates as photoactive materials for optoelectronic devices. Here, photodetectors based on mechanically exfoliated ReSe_2_ crystals were fabricated using chemical vapor deposited (CVD) graphene single-crystal (GSC) as lateral contacts. A “pick & place” method was adopted to transfer the desired crystals to the intended position, easing the device fabrication while reducing potential contaminations. A similar device with Au was fabricated to compare contacts’ performance. Lastly, a CVD hexagonal boron nitride (hBN) substrate passivation layer was designed and introduced in the device architecture. Raman spectroscopy was carried out to evaluate the device materials’ structural and electronic properties. Kelvin probe force measurements were done to calculate the materials’ work function, measuring a minimal Schottky barrier height for the GSC/ReSe_2_ contact (0.06 eV). Regarding the electrical performance, I-V curves showed sizable currents in the GSC/ReSe_2_ devices in the dark and under illumination. The devices presented high photocurrent and responsivity, along with an external quantum efficiency greatly exceeding 100%, confirming the non-blocking nature of the GSC contacts at high bias voltage (above 2 V). When introducing the hBN passivation layer, the device under white light reached a photo-to-dark current ratio up to 10^6^.

## 1. Introduction

Transition metal dichalcogenides (TMDCs) are semiconductors of the MX_2_ type, where M is a transition metal (e.g., molybdenum, rhenium, tungsten) and X is a chalcogen element (e.g., sulfur, selenium, and tellurium). This class of layered materials can be produced in two-dimensional (2D) form or thinned down to atomic thickness, i.e., by exfoliating the corresponding bulk crystals that have weak interlayer van der Waals bonding [1,2]. Two-dimensional TMDCs are structurally stable materials that exhibit interesting electronic and optical properties: direct and tunable bandgap, considerable exciton binding energy, high carrier mobility, strong spin-orbit coupling, strong photoluminescence (especially in monolayer form), and nonlinear optical properties [1,2]. As such, 2D TMDCs find potential applications in electronics, optoelectronics, photonics, sensing, and energy storage [3,4,5,6,7]. A key characteristic of atomically thin TMDCs is the possibility to select or grow crystals with a specific number of layers, thus tuning the optoelectronic properties to comply with device requirements [3]. However, this can translate into technical difficulties related to thickness control and sample uniformity. Recent studies on 2D rhenium-based dichalcogenide materials–ReX_2_ (X = S or Se)–have shown optoelectronic properties that are independent of the number of layers: Even in bulk, the layers act as electronically and vibrationally decoupled monolayers [8,9]. These features give ReX_2_ an excellent potential for optoelectronic nano-devices and 2D heterostructures, releasing device fabrication and uniformity control constraints. For these reasons, and owing to their structural and electronic properties [9,10,11,12,13,14], ReX_2_ has attracted significant scientific and technological interest [10,12,13,15]. In particular, ReSe_2_ has a bandgap of ~1.3 eV [9] and is suitable for optoelectronic applications, although only a few groups tested it in transistors and photodetectors [15,16,17,18,19].

In the last years, several 2D TDMCs configurations (e.g., so-called van der Waals heterostructures) were proposed to improve the performance or unlock new functionalities in optoelectronics and spintronics [1,3,4,5,6]. Numerous approaches were proposed to make 2D heterostructures: mechanical exfoliation and sequential layer restacking, layer-by-layer chemical vapor deposition (CVD), or any combination of these approaches, each presenting its pros and cons. Mechanical exfoliation is a simple technique that can be coupled with the stamping method with the aid of polymeric viscoelastic stamps (e.g., PDMS) [20]. The device fabrication is quite versatile, but the throughput is extremely low. By contrast, CVD is suited for each layer’s direct growth but requires rigorous control and optimization of the growth parameters and cannot grant control on the layer positioning and geometry: post-processing is thus required, which adds complexity to the whole device fabrication process. When making 2D TMDC-based electronic devices, it became apparent that input/output metallic contacts may introduce severe technical challenges [21,22,23,24]. A non-ohmic contact at a mismatched metal-semiconductor interface forms a Schottky barrier that can impact the device’s functionality and performance. Consequently, the choice of optimal electrical contacts is crucial [24,25], as is the realization of ultra-clean interfaces among 2D TDMCs [26]. The properties of 2DMs are dependent on the surface chemistry and interface coupling to a large extent, so clean interfaces between different 2DMs are vital to maximizing the device performance [27]. As a contact material, graphene has a high thermal conductivity and electron mobility, but in polycrystalline graphene (i.e., the most common form) the transport largely depends on the grain boundary concentration [28]. The realization of graphene contacts is usually a versatile and non-damaging process that provides defect-free interfaces [29]. Graphene is often coupled with hexagonal boron nitride (hBN) layers, serving as a substrate or encapsulating layers, maximizing graphene’s electronic performance. hBN is a large bandgap insulator (5.97 eV) [30], chemically inert, and free of dangling bonds or surface charge traps. It has a low lattice mismatch with graphene (1.7%) [31] providing effective passivation (e.g., on top of Si/SO_2_ or as an interlayer in 2D van der Waals heterostructures) [32].

In this work, we fabricate photodetectors based on mechanically exfoliated ReSe_2_ crystals. A “pick & place” method is adopted to (i) allow the identification of the most fitting ReSe_2_ crystals among the wide range of exfoliated sizes and (ii) transfer the selected crystals to the desired location. Since this is an all-dry procedure, the possibility of additional contaminations is reduced. Using this technique, the semiconductor is interfaced with CVD GSC contacts in a lateral-heterostructure design. ReSe_2_ serves as an efficient photoactive layer, while graphene can collect the charge carriers without introducing a sizeable Schottky junction at the interface [33]. A similar device with Au/ReSe_2_ is fabricated to compare Gr and Au contacts’ performance. Building on this design, a CVD hBN passivation layer is introduced to fabricate an hBN/GSC/ReSe_2_ device with higher photoactive layer thickness. Raman spectroscopy of the device materials is carried out to evaluate the materials’ structural and electronic properties. Kelvin probe force measurements (KPFM) are conducted to analyze and compare the GSC/ReSe_2_ and Au/ReSe_2_ devices’ topography and work functions. To evaluate the electrical performance of the devices, I-V curves were acquired in the dark and illumination. The Au/ReSe_2_ and GSC/ReSe_2_ devices are tested under an intense white light to measure the photoconductivity as a function of the incident optical power by filtering the light beam with different neutral density filters, and also under 530 and 790 nm illumination, to calculate the external quantum efficiency (EQE). To study the hBN/GSC/ReSe_2_ device photoconductivity, time-resolved photoresponse is measured with a white light illumination and decay measurements are performed at increasing bias voltage.

## 2. Materials and Methods

### 2.1. CVD Growth and Transfer of Graphene and hBN

Graphene single crystals were grown on Cu foils via low-pressure CVD. A Cu foil substrate (25-µm-thick, Alfa Aesar, purity 99.8%), serving as a metal catalyst, was cleaned by an ultrasonic bath in a mixture of 20 mL 0.5 M FeCl_3_, 20 mL 37% HCl and 360 mL de-ionized (DI) water, followed by rinsing in DI water. The Cu substrate was pre-oxidized by placing the foil onto a hotplate at 200 °C for 30 min. The Cu substrate is then loaded in a height-controlled sapphire cavity, placed in a 10 cm × 10 cm graphite box and loaded in the CVD tube. The substrate was annealed at 1040 °C for 30 min in Ar atmosphere (500 sccm, 9 Torr). For graphene growth, the gas mixture is switched to Ar/H_2_/CH_4_ (250/100/1.2 sccm, 4 Torr) keeping the temperature of 1040 °C for 40 min (temperature oscillations <0.5 °C). After the growth, the tube is cooled down to room temperature in Ar (500 sccm). hBN films were grown on Cu foil substrates in a CVD system with an independent pre-heating reservoir cell and an injector to deliver the precursor vapor over the substrate. 300 mg of ammonia borane (AB, H_3_BNH_3_, Sigma-Aldrich, St. Louis, MO, USA) were loaded in the reservoir cell and pre-heated at 80 °C (at 3 °C/min) for 4 h in H_2_ atmosphere, and then let cool down to room temperature (at 3 °C/min). The pre-heating step serves to decompose AB into diammoniate of diborane ([H_2_B(NH_3_)_2_][BH_4_], DADB), suppressing the melting point and allowing for a shorter deposition time under more stable growth conditions. The precursor vapor mixed to H_2_ was allowed in the CVD tube where an Ar/H_2_ mixture (200/50 sccm, at 2 Torr) carried it to the Cu substrate. For the growth of the hBN film, the temperature was set at 1000 °C for 15 min. After cooling down and extraction, the films were transferred onto Si/SiO_2_ wafers by PMMA-assisted wet transfer [34].

### 2.2. Devices Fabrication

**Figure 1:**
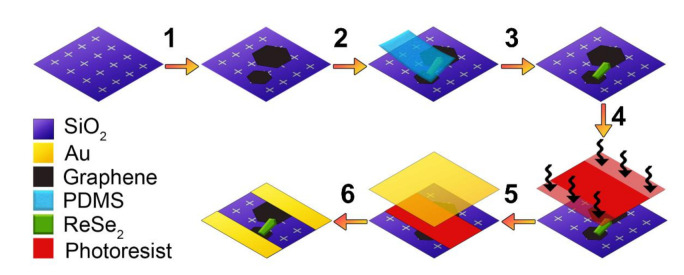
Schematic of the GSC/ReSe_2_ device production process: (1) transfer of GSC to a Si/SiO_2_ substrate with crosshair markers, (2–3) placement of the exfoliated ReSe2 crystal across two discrete GSCs by “pick & place” method, using a PDMS stamp, (4) spin-coating of photoresist and exposure with laser lithography to form the mask for the metallic contacts, (5) Cr/Au layers sputtering, and (6) partial removal of the metallic layer (lift-off) to form the Cr/Au contacts.

The fabrication of the GSC/ReSe_2_ lateral-heterostructure device was carried out as follows. CVD-grown GSCs were transferred to a Si/SiO_2_ substrate with TiWN crosshair markers by a similar PMMA-assisted wet transfer method as stated in ref. [34]: PMMA was spin-coated on Cu/GSCs as a sacrificial layer, and FeCl_3_ (0.5M) was used as Cu etchant. After a 2 h etch, the PMMA/GSCs stack was cleaned in deionized water (DIW) three times before transferring to the final substrate, drying, and finally removing the PMMA (in acetone bath overnight at room temperature). Couples of neighboring GSCs suitable for the device fabrication were identified at the microscope and localized using the markers. An n-type ReSe_2_ crystal (produced by M. Pumera’s group [35]) was then placed across two neighboring GSCs to form a channel, using the “pick & place” method, described as follows. After mechanical exfoliation with Nitto tape (SPV 224), the ReSe_2_ crystal was transferred to a viscoelastic PDMS (PF Gel-film from GelPak, Hayward, CA, USA). The crystal thickness and homogeneity were assessed at the microscope by evaluating the optical contrast. After identifying the desired crystal, it was transferred across the two GSCs on the Si/SiO_2_ substrate. The PDMS + ReSe_2_ stack was then mounted on a glass slide and attached to an XYZ micromanipulator, with the crystal facing the substrate (fixed on an XY axis movable stage). Since the stamp was transparent, the microscope could be used to find and align the ReSe_2_ crystal to the identification markers on the substrate for it to bridge exactly the two neighboring GSCs. The stamp was lowered to get in touch with the substrate and gently pressed with a cotton swab. This allows an optimal contact between stamp and substrate, making sure that no air bubbles are trapped beneath the crystal. Finally, the stamp was slowly raised using the micromanipulator while monitoring the peeling process with the microscope. Images with an optical microscope were taken and used as a template for the Au contacts’ mask drawing to have pads accessible to conventional tips for the electrical measurements. The substrate was spin-coated with AZ 1505 photoresist (Microchemicals, Ulm, Germany) and then soaked on a 4:3 aqueous TMAH-based solution (AR 300-47, Allresist GmbH, Strausberg, Germany). This process induces partial insolubilization of the photoresist surface. It creates an overhang after exposure with laser lithography (DWL 2000, Heidelberg Instruments, Woburn, MA, USA) and development (AZ 400K developer 1:4, AZ Electronic Materials USA Corp., Somerville, NJ, USA) that ensures good metallic contact profiles and an effective lift-off process. A 3/50 nm thick Cr/Au layer was sputtered with a Kenosistec UHV sputtering tool and the metallic layer was removed (lift-off) by a room temperature acetone bath overnight. The entire device fabrication process schematic is shown in Figure 1.

An analogous Au/ReSe_2_ device was fabricated to compare the performance of graphene and Au contacts. A total of 3/50 nm Cr/Au contacts were sputtered onto a Si/SiO_2_ (without markers) by the same process as explained above to form two 400-μm-wide electrodes with a 3-μm-gap separating them. Subsequently, an exfoliated ReSe_2_ was transferred to bridge the two Au contacts by the “pick & place” method.

**Figure 2:**
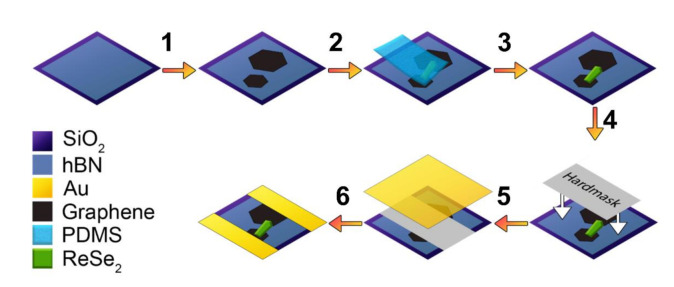
Schematic of the hBN/GSC/ReSe_2_ device production process: (1) transfer of GSC to a Si/SiO_2_/hBN substrate, (2–3) placement of the exfoliated ReSe_2_ crystal across two discrete GSCs by “pick & place” method, using a PDMS stamp, (4) placement of a glass shadow mask (125-μm-thick) to form the mask for the metallic contacts, (5) Cr/Au layers sputtering, and (6) removal of the hard mask to form the Cr/Au contacts.

An hBN/GSC/ReSe_2_ lateral-heterostructure device including a CVD hBN passivation layer was also fabricated to test our geometry with a thicker ReSe_2_ crystal. The hBN film (4 nm thick) was transferred onto a Si/SiO_2_ substrate (without markers) to act as a 2D passivation layer and a substrate for graphene. GSCs were transferred onto the Si/SiO_2_/hBN substrate. Two GSCs with lateral sizes of ~250 µm and ~510 µm, spaced by 135 μm were identified at the microscope. A ReSe_2_ crystal (600-nm-thick) was picked and placed across the two GSCs establishing a channel. The Cr/Au electrodes were fabricated by sputtering via a 125-μm-thick glass hard mask. The device fabrication process is schematically shown in Figure 2.

### 2.3. Raman Spectroscopy

Measurements were carried out on an ALPHA300 R Confocal Raman Microscope (WITec) using 532 nm laser light for excitation in the backscattering geometry at room temperature (the laser beam was focused on the sample by a 100× lens–Zeiss). The mapping measurements were performed using a 600 groove/mm grating with P_Laser_ = 0.8 mW, 4 s acquisition time and a 0.5 μm/point resolution. Using the WITec Suite FIVE software and the TrueComponentes analysis tool, three different spectra corresponding to each component-SiO_2_ substrate, ReSe_2,_ and GSC-were selected and automatically color-coded.

### 2.4. Atomic Force Microscopy (AFM)

Bruker Dimension ICON in tapping-mode was used with NANOSENSORS™ PPP-NCH tips with an average tip radius of curvature <10 nm, force constant of 10–130 N/m, and a 258.12 kHz resonance frequency. Image analysis was performed with Gwyddion software. To calculate the active area of the devices, S, only the region of the ReSe_2_ flakes in between the contacts was selected.

### 2.5. Kelvin Probe Force Microscopy (KPFM)

Kelvin probe force microscopy (KPFM) experiments were performed in the same Bruker Dimension Icon AFM operated in air, using the amplitude modulation mode. For the dual-pass method, the lift height was set to 10 nm. We used Pt/Ir coated cantilevers (PPP-EFM Nanosensors, Neuchatel, Switzerland) with a nominal tip radius of 25 nm, a spring constant of 2.8 N/m, and 75 kHz resonance frequency. The tips were calibrated using a Au-coated Si sample for comparability of results. The contact potential difference (CPD) is defined as V_CPD_ = e(Φ_tip_ − Φ_sample_), where Φ is the work function.

### 2.6. Electrical Measurements

The Seebeck coefficient of the ReSe_2_ flake semiconductor on Au contacts was measured by heating the positive contact while keeping the negative contact at room temperature. Electrical properties of all three types of devices (Au/ReSe_2_, GSC/ReSe_2_ and hBN/GSC/ReSe_2_) were measured with a source meter (Keithley 6470, Keithley Instruments, Solon, OH, USA) under ambient conditions. A different Au/ReSe_2_ device from the KPFM measurements was used for electrical characterization, but its structure and contacts are the same, while having a similar ReSe_2_ flake thickness within the same bulk regime (58 nm). For both the Au and the GSC devices I-V curves were acquired in the dark and under white light illumination for voltages between −2 and 2 V. The devices were also measured under 530 and 790 nm illumination and applied bias $V_{\mathrm{bias}}=2\;V$, in order to calculate the responsivity and external quantum efficiency (EQE). The photoconductivity was measured as a function of the incident optical power by filtering the incident light beam with different neutral density filters. As for the hBN/GSC/ReSe_2_ device, decay measurements were performed at increasing bias voltage: 50, 80, and 100 V. A 250 W halogen lamp with a spot size of 30 μm in diameter delivering an optical power of 962 W/m^2^ was used. An external bias voltage of V_bias_ = 50 V applied between contacts was used for all the experiments under illumination.

## 3. Results

**Figure 3:**
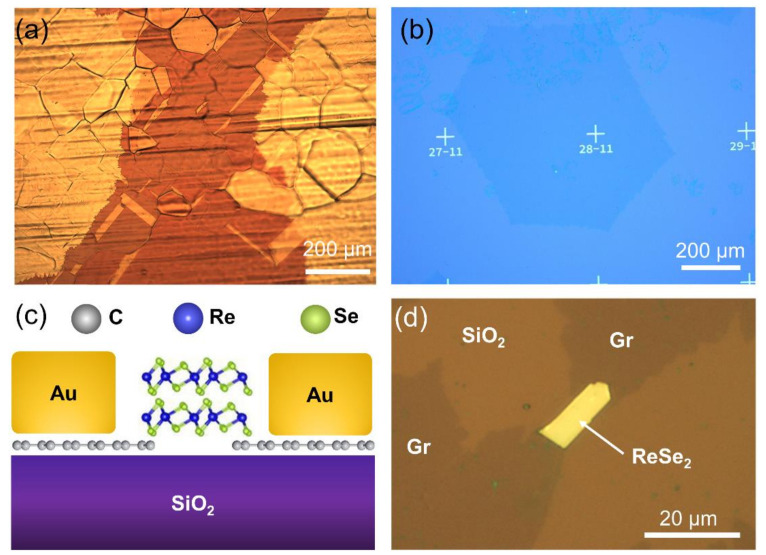
(**a**) GSCs grown by CVD on Cu substrates, which was oxidized after growth to enhance the contrast between GSC and substrate (**b**) The GSCs were transferred onto Si/SiO_2_ wafer. (**c**) Schematic side-view and (**d**) optical top-view image of the GSC/ReSe_2_ photodetector device. The ReSe_2_ channel has ~5 μm width.

As a first step, we optimized the CVD process to produce sub-mm GSCs used as device contacts. The Cu substrates were pre-oxidized to partially passivate the Cu surface and decrease the graphene nucleation sites [36]. The pre-oxidation also enables the progressive release of a stable oxygen supply from the substrate during the high-temperature growth (1040 °C) and increases the crystal growth rate. The sapphire cavity also releases trace amounts of oxygen that stabilize the Cu oxidation level, compensating for the reduction of Cu-O bonds by atomic hydrogen. The graphite box mimics the Cu-enclosed configurations reported in the literature [37], by increasing the Cu vapor pressure inside the cavity and thus reducing the substrate roughness (due to continuous Cu re-deposition). By this approach, we could control the GSC size from 50 μm to a few millimeters. By keeping the initial seed concentration low and tuning the deposition time, the GSCs do not coalesce and can grow to sub-mm sizes and beyond, as shown in Figure 3a. For the larger crystals, the graphene nucleation density was as low as <600 nuclei/cm^2^. Unlike the Cu-enclosed configuration, our approach does not damage the catalyst substrate and allows upscaling graphene growth on flat Cu foils of arbitrary size. After the transfer to a SiO_2_ substrate (Figure 3b), two GSCs of ~0.25 mm^2^ size separated by a small gap of ~5 μm were identified. As detailed in the Section 2.2, the GSC/ReSe_2_ photodetector device studied in this work is a two-terminal photoresistor with in-plane current flowing via a ReSe_2_ channel across two GSC contacts (acting as source and drain). The device is depicted conceptually in Figure 3c and shown in Figure 3d.

**Figure 4:**
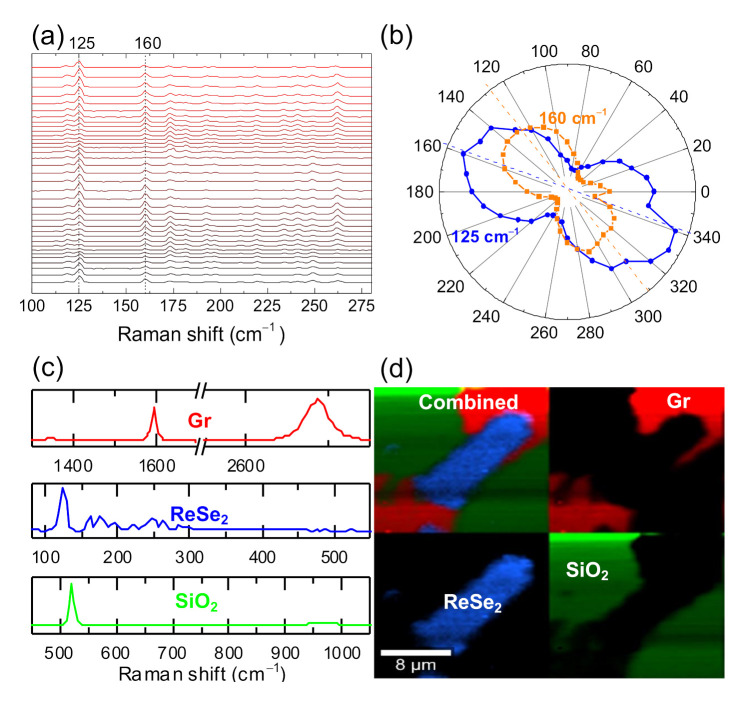
(**a**) Incident light polarization-dependent Raman spectra, obtained by rotating the sample from 0° to 360° (with a step of 10°) and the initial angle being arbitrary to the sample crystallographic axis. The peaks at 125 cm^−1^ and 160 cm^−1^ are strongly related to the rotation sample angles. (**b**) A plot of intensities of the two modes (125 cm^−1^ mode in dark blue and 160 cm^−1^ mode in orange). (**c**) Raman spectra representative of ReSe_2_ (blue line), graphene (red line) and SiO_2_ (green line). (**d**) Color-coded Raman map of the device, combining SiO_2_ (green), GSC (red), and ReSe_2_ (blue) Raman spectra. The Raman spectra presented in (**a**) were used as a reference to obtain the Raman mapping images.

Raman spectroscopy was performed to evaluate the quality and structural properties of the 2D materials. The Raman spectra collected on the ReSe_2_ crystal show an intricate signature in the 100–300 cm^−1^ range, with 13 first-order Raman active modes. This complex spectrum is attributed to the low crystal symmetry of Re-based dichalcogenides (associated with fundamental modes coupled to each other) and acoustic phonons [10]. Two prominent peaks at ~125 and ~160 cm^−1^ (Figure 4a), assigned to E_g_-like and A_g_-like modes, respectively [14], were chosen to probe the anisotropic crystalline structure of ReSe_2_ and study the dependence of the Raman modes to the laser light angle (by rotating the sample between 0–360°) [14,38]. Figure 4a shows that the E_g_-like (125 cm^−1^) and A_g_-like (160 cm^−1^) mode intensities strongly depend on the rotation angles, confirming the anisotropy of the ReSe_2_ structure. The integrated intensities of these two modes are plotted in Figure 4b and are in line with previous studies [14]. The *b*-axis of the ReSe_2_ lattice, which is along the Re_4_ chains in the crystal, is parallel to the longer axis (dashed blue line in Figure 4b), connecting the two E_g_-like mode maximum intensity points. Figure 4c shows the Raman spectra of Si/SiO_2_, graphene, and ReSe_2_ separately. In the Si/SiO_2_ spectrum, the usual peak at 521 cm^−1^ due to the Si (001) substrate dominates. The I_2D_/I_G_ ratio for graphene, the minimal D band, and a sharp symmetric 2D peak indicate high-quality defect-free single-layer graphene [39,40,41,42]. This is further supported by the 2D peak analysis, reporting a single and sharp (FWHM~31.9 cm^−1^) Lorentzian band centered at ~2686.5 cm^−1^ [39,43]. Figure 4d shows a color-coded Raman map of the device, build with the spectra shown in 4c. Each pixel in the image stores one full Raman spectrum, and it is colored according to its similarity to one or more spectra in Figure 4c. All the images are consistent and give an accurate surface description based on the chemical composition of the area mapped.

**Figure 5:**
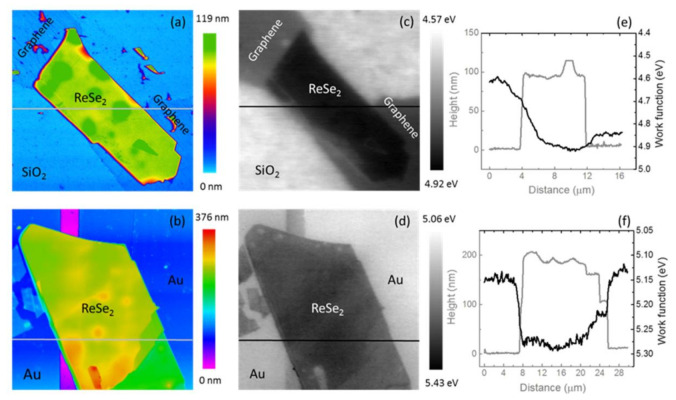
KPFM characterization of ReSe_2_ devices. (**a**,**b**) Tapping mode topography (16.5 µm × 16.5 µm and 30 µm × 30 µm) of ReSe_2_/graphene/SiO_2_ and ReSe_2_/Au. (**c**,**d**) Surface work function maps of the two devices. (**e**,**f**) Height and work function line profiles were obtained along the indicated gray and black solid lines. The right axis represents the absolute value of the work function.

Kelvin probe force microscopy measurements were conducted to evaluate the SBH between ReSe_2_ and two different sets of metallic contacts, namely graphene and Au. Topography and work function maps are shown in Figure 5a–d. For the GSC/ReSe_2_ device, the profiles in Figure 5e indicate a thickness of ~95 nm and a work function Φ ≈ 4.92 ± 0.02 eV for the ReSe_2_ crystal, in good agreement with previously reported values [44]. The SBH_GSC_ was calculated from the work function difference between GSC contact and ReSe_2_ considering the average of several profiles measured, obtaining SBH_GSC_ ≈ 0.06 eV [45]. For the Au/ReSe_2_ device, the ReSe_2_ has a thickness ~180 nm and a work function Φ ≈ 5.25 ± 0.02 eV (Figure 5f), giving SBH_Au_ ≈ 0.15 eV.

**Figure 6:**
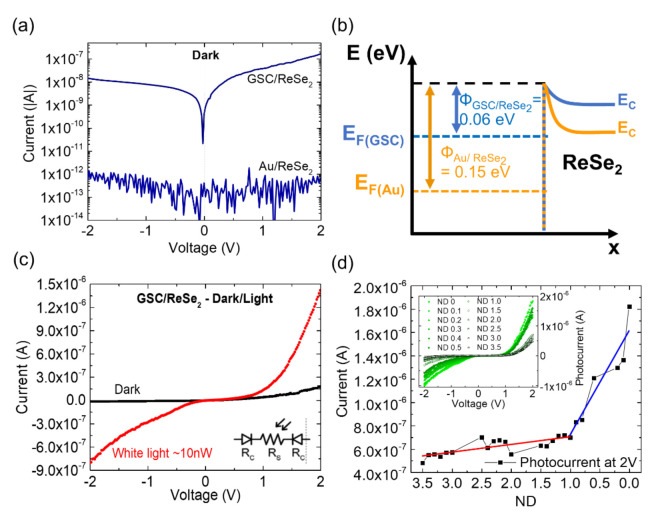
(**a**) I-V curves comparing GSC/ReSe_2_ and Au/ReSe_2_ devices without light illumination. (**b**) Band diagram of GSC/ReSe_2_ and Au/ReSe_2_ devices based on the KPFM results. (**c**) Dark and light I-V curves of the GSC/ReSe_2_ device and the equivalent circuit with two Schottky barriers connected back-to-back with a series resistance (inset). (**d**) Response of the same device under white light and different attenuator filters (inset) and the corresponding current as a function of attenuator filters to an applied bias of 2 V highlighting the two different linear regimens (red and blue lines) with a transition point at ND~1.

The ReSe_2_ flake Seebeck effect $\left(\alpha_{n}=-\frac{\Delta V}{\Delta T}\right)$ was measured [46], obtaining a negative Seebeck voltage, concluding that the semiconductor is n-type ($\alpha_{n}<0$). The electrical characteristics were further studied to understand the device’s transport mechanism, and the results are shown in Figure 6. Figure 6a compares the I-V curves of the GSC/ReSe_2_ and Au/ReSe_2_ devices in the dark. The GSC/ReSe_2_ device presents consistently higher dark currents. Figure 6b shows a band diagram of GSC/ReSe_2_ and Au/ReSe_2_ contact junctions based on KPFM results. An equivalent circuit with two diodes connected in a back-to-back configuration with a series resistance (see Figure 6c inset) describes the Schottky barriers in the device. Figure 6c shows the I-V curves in the dark and under white light illumination. Figure 6d shows room temperature photocurrent values at V_bias_ = 2 V as a function of incident white light power by filtering the incident beam with neutral density filters of decreasing number, *ND*, from *ND* = 3.5 to 0 (no filter). Two different linear regimes were identified, one with a supralinear behavior (marked by a red line) and the other with an approximately linear trend (marked by a blue line).

**Figure 7:**
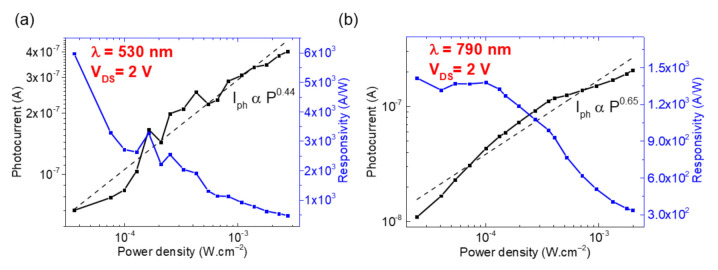
Photocurrent and responsivity as a function of irradiance under two different wavelengths exhibiting a P^α^ trend (**a**) 530 nm with a fit line for α = 0.44 and (**b**) 790 nm with a fit line for α = 0.65 for GSC/ReSe_2_ device.

The GSC/ReSe_2_ device photocurrent (I_ph_ = I_light_ − I_dark_) was measured under 530 nm and 790 nm wavelength illumination (Figure 7). It is worth noting that the measured photocurrent is the secondary photocurrent, which allows for a maximum gain greater than unity. The photocurrent increases with the incident illumination density, showing a power-law behavior $\left(I_{\mathrm{ph}}\propto {\;P}^{\alpha_{\lambda }}\right)$, with exponent α_530nm_ = 0.44 and α_790nm_ = 0.65. At V_bias_ = 2 V, the photocurrent is ~0.4 μA and ~0.21 μA, respectively. The responsivity $\left(R_{\lambda }=\frac{I_{\mathrm{ph}}}{P\times S}\right)$ and external quantum efficiency $\left(\mathrm{EQE}\;=\frac{{\mathrm{hcR}}_{\lambda }}{e\lambda }\right)$ were also calculated (P is the optical power density, S the active illuminated area of the device, being *S* = 34 μm^2^). The responsivity at maximum illumination power (P_530nm_ = 2.8 × 10^−3^ W·cm^−2^, P_790nm_ = 2.0 × 10^−3^ W·cm^−2^ is R_530nm_ = 4.7 × 10^2^ A/W and R_790nm_ = 3.3 × 10^2^ A/W. In terms of EQE, the calculated values are EQE_530nm_ = 1090% and EQE_790nm_ = 518%.

**Figure 8:**
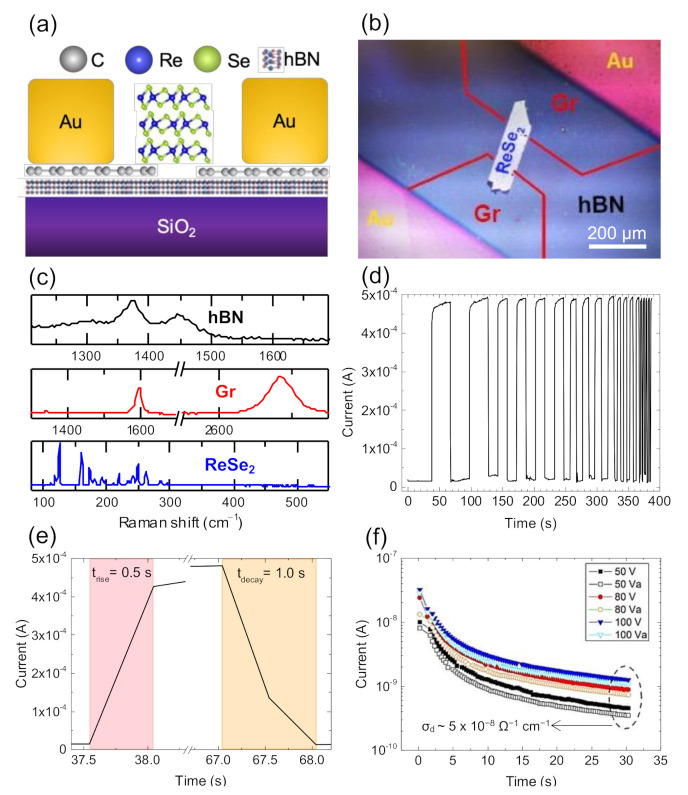
(**a**) Schematic side-view and (**b**) optical top-view image of the GSC/ReSe_2_ device with the hBN passivation layer. (**c**) Raman spectra representative of hBN (black line), graphene (red line), and ReSe_2_ (blue line). (**d**) Time response under illumination at V_bias_ = 50 V and (**e**) rise/decay time plot. (**f**) Photoresponse decay was performed at three increasing bias voltages: V_bias_ = 50, 80, 100 V. V_a_ is the second scan, obtained immediately after the first scan.

Another ReSe_2_-based photodetector was fabricated by interfacing CVD-grown GSCs with an hBN passivation layer (see Section 2). For this device, a thicker ReSe_2_ crystal (~600 nm) was exfoliated and selected to act as an absorbing layer, the purpose of testing the graphene contacts under much higher current levels. The schematic of the hBN/GSC/ReSe_2_ device is depicted in Figure 8a, while an optical image is presented in Figure 8b. The materials were analyzed by Raman spectroscopy (Figure 8c), with results in line with the previous device: a 2D peak centered at ~2686.5 cm^−1^ for crystalline, monolayer graphene; two prominent peaks at ~125 and ~160 cm^−1^ for the mechanically exfoliated ReSe_2_ crystal. The analysis of the CVD hBN confirms the expected E_2g_ peak at ~1372 cm^−1^, corresponding to crystalline, few-layer hBN [47]. Photocurrent measurements (Figure 8d–f) were conducted on this hBN/GSC/ReSe_2_ device. Figure 8d shows the time-resolved photoresponse obtained with a white light illumination through a chopper, with a high V_bias_ = 50 V to guarantee efficient photon absorption through most of the ReSe_2_ thickness. The photocurrent oscillations follow the on/off cycles set by the chopper within the range of frequencies studied. The rise and decay times in each cycle were calculated by measuring the step time (Figure 8e), obtaining τ_rise_ = 0.5 s and τ_decay_ = 1.0 s. In Figure 8f, the photocurrent decay is recorded after turning off the light at t = 0 s, under the applied bias of V_bias_ = 50, 80 and 100 V. Each measurement under constant bias was repeated immediately after the first scan. The photocurrents show an exponential decay with a characteristic decay time τ_decay_ = 2.2 s, before reaching a steady-state value, which corresponds to the dark current. In order to compare the device performances, we calculated the photoconductivity under white light illumination ($\sigma_{\mathrm{ph}}=\frac{I_{\mathrm{ph}}}{A\times F}$, where $I_{\mathrm{ph}}$ is the measured photocurrent, A the cross-sectional area of the ReSe_2_ flake and F the applied electric field—$F=\frac{V}{L}$, with V being the applied voltage and L the ReSe_2_ flake length), as shown in Table 1.

As a first remark, the photoconductivity increases by three orders of magnitude when the GSC contacts are introduced. Notwithstanding the thicker ReSe_2_ crystal, the hBN/GSC/ReSe_2_ device photoconductivity is not so further increased, pointing at the low impact of the flake thickness on the overall device performance.

## 4. Discussion

The direct deposition of metallic contacts on atomic-thick materials (such as 2D TMDCs) can be detrimental, easily breaking in-plane covalent bonds and degrading the device performance [23]. By contrast, graphene provides advantages over standard metals in terms of formation of clean and defect-free interfaces through a non-damaging process [28,29]. Due to its very high thermal conductivity, graphene can also act as an excellent heat sink, reducing the chance of localized heat-induced damage to 2D semiconductors at high current densities, thus increasing the device’s long-term stability [21]. When a metal contact is deposited on a semiconductor, a Schottky barrier may form depending on the metal’s work function and the semiconductor electron/hole affinity [48]. In general, a sizable Schottky barrier height (SBH) leads to a rectifying contact that limits the current injection/extraction, as opposed to ohmic contacts (setting no barrier to the current flow) [25]. In 2D materials, the absence of dangling bonds on either side of the in-plane conduction path and the anisotropic charge carrier transport across the active region can give rise to very high contact resistance, due to large SBH or Fermi level pinning [49,50]. Thus, the appropriate material choice for low-dimensional contacts is essential to prevent or minimize the SBH [21,51]: metal/graphene/TMDC contacts showed ohmic-like behavior due to minimal SBH [52,53]. Furthermore, it is possible to tune the work function of graphene in 2D devices by external gating [54,55]. In our devices with back-to-back configuration, the reverse saturation current (I_s_) of each diode (formed at the junctions between the two metal contacts and ReSe_2_) exponentially decay with the Schottky barrier height (Φ*_SHB_*), as follows:

(1) $$I_{s}\;\propto \;\exp\left(-\frac{q\Phi_{SHB}}{k_{B}T}\right)$$

As shown in Figure 6a, the GSC/ReSe_2_ dark current is ~160 nA (at 2V), in line with previous reports on undoped ReSe_2_ devices, usually indicating low carrier density (~3 × 10^13^ cm^−2^ [56]). The ReSe_2_ resistivity was 17 Ω∙cm (close to ~5 Ω∙cm reported in ref. [57]). The Au/ReSe_2_ dark current was ~0.5 pA, i.e., 3.2 × 10^5^ smaller than the graphene-contacted devices. The device dark currents can be thus easily explained by Equation (1), when considering the two Φ*_SHB_* measured by KPFM (i.e., 0.06 eV for GSC, 0.15 eV for Au), as illustrated by the band diagrams in Figure 6b. The small electron barrier in the GSC/ReSe_2_ device increases the probability of electron injection from the metal to the ReSe_2_ film even at room temperature. In the GSC/ReSe_2_ device under illumination, a marked increase in ReSe_2_ conductivity was observed (Figure 6c). At low bias (V_bias_ < 1.5 V), the current limiting effect of the two Schottky barriers on the current is highlighted by the non-linear IV characteristic. For higher bias (~1.5 V < V_bias_ < 2 V), the contacts appeared as non-blocking, with the current showing linear behavior [58]. The room temperature measurements at V_bias_ = 2 V (Figure 6d) showed a photocurrent trend following two regimes: a supralinear behavior (marked by the red line) around a transition point, occurring at *ND* > 1, and an approximately linear trend (marked by the blue line) for *ND* < 1 (see also the inset). This behavior could be explained by a combination of effects due to sensitization and electronic doping. Increased doping can occur by increasing the light intensity, which generates higher photocarrier concentrations. Changing carrier concentration is equivalent to moving the steady-state pseudo-Fermi levels (*E_Fn_* and *E_Fp_*) across the semiconductor bandgap. This transformation occurring for *ND* < 1 and entirely changes the carrier transport as the localized states inside the bandgap change from traps to recombination centers as *E_Fn_* (*E_Fp_*) move up (down) in the semiconductor bandgap, scanning different defect bands [59].

The photocurrent (I_ph 530nm_ = 0.4 µA, I_ph 790nm_ = 0.21 µA) of our GSC/ReSe_2_ device was one order of magnitude higher than those reported for similar ReSe_2_ devices with metallic contacts (see Table 2). The photocurrent increases with the incident illumination density, showing a power-law behavior ($I_{\mathrm{ph}}\propto {\;P}^{\alpha_{\lambda }}$) with α_530nm_ = 0.44 and α_790nm_ = 0.65. A value close to 1 indicates a photoconductive response, while 0 < α_λ_ < 1 indicates a dominant photogating behavior, as expected for low-dimensional photodetectors with very high exciton binding energy and reduced dielectric screening [60,61]. The calculated responsivity with maximum illumination power (P_530nm_ = 2.8 × 10^−3^ W.cm^−2^ and P_790nm_ = 2.0 × 10^−3^ W.cm^−2^) is R_530nm_ = 4.7 × 10^2^ A/W and R_790nm_ = 3.3 × 10^2^ A/W are superior to the second device we fabricated with Au contacts (flake thickness of 58 nm and S = 26 μm^2^) and to other works on exfoliated ReSe_2_ devices. Furthermore, these values are similar to those obtained for a p-doped ReSe_2_ device fabricated [62] (see Table 2). The secondary photocurrent measure depends on the majority carrier concentration or on the photocarrier with the higher mobility, μ. Therefore, it is possible to have gain, G, given by the ratio of photocarrier lifetime, τ_ph_, to photocarrier transit time, τ_tr_ (G = τ_ph_/τ_tr_) [59]. Then, the calculated EQE values (EQE_530nm_ = 1090% and EQE_790nm_ = 518%) are >>100% and vastly superior to the Au/ReSe_2_ device. These values confirm the non-blocking nature of the graphene contacts at the working bias, result in a photoconductive gain [59]. Such large EQE values may be due to a distribution of traps that decreases the lifetime of one carrier type while increasing the other’s (i.e., sensitization), with the photoconductive gain being proportional to the lifetime of the photo-excited carriers [63,64]. To compare our GSC/ReSe_2_ device with other reported works, Table 2 summarizes the parameters and performance of ReSe_2_-based photodetectors. Recent progress in CVD-grown ReSe_2_ devices [38,65] has shown a lower photo-responsivity (between ~3–8 A/W) compared to other devices with exfoliated crystals. Further, a wide range of mobility values is observed, from 9.8 to 1.36 × 10^−3^ cm^2^/(V.s), primarily due to a variable density of grain boundaries in polycrystalline CVD samples. Similar works with exfoliated ReSe_2_ devices with Au electrodes reported <100 nA photocurrents and lower responsivities [12,66], identical to the CVD-grown ReSe_2_ devices.

In electronic devices, graphene can be strongly influenced by the underlying substrate. Although still the ubiquitous choice, SiO_2_ substrates for 2DMs may present several constraints related to roughness [71], charged impurities [30,72,73], and surface optical phonons [73,74]. Consequently, electron-hole charge fluctuations can originate in graphene on SiO_2_, scattering charge carriers and hence limiting device performance [30,75]. Graphene can reach the highest electron mobility and show minimal carrier inhomogeneity when supported by hexagonal boron nitride (hBN) [32,76]. hBN’s surface optical phonon modes have higher energies than similar modes in SiO_2_, improving high-temperature and high-electric-field performance of hBN and graphene-based devices [77,78]. Moreover, the inclusion of an hBN layer in the device can reduce roughness, intrinsic doping, and chemical reactivity of the fabricated device [71]. Following this rationale, we tested the introduction of an hBN passivation layer onto SiO_2_. For this device, a ReSe_2_ flake with a higher thickness (600 nm, about ×6 the previous one) was selected to demonstrate the thickness-independence of the ReSe_2_ optoelectronic properties while maximizing the photon harvesting and increasing the signal-to-noise sensor output. Notice that photocurrent levels (Figure 8d,e) are approximately two orders of magnitude higher (I_ph_ ~10^−4^ A) than in the previous experiments (I_ph_ ~10^−6^ A), as expected for a thicker ReSe_2_ absorbing layer and a higher applied voltage. Even so, the graphene-on-hBN contacts provided a sufficient current injection and collection able to sustain the photocurrent in all cases. In photoresponse decay measurements (Figure 8f), a steep current decay was observed. The decay time corresponds to the transient regimen between the two steady states—under illumination and in the dark—during which carriers de-trap and drift to the contacts under the applied field. The measured decay time, extracted from the fitting to an exponential profile, is τ_decay_ = 2.2 s in line with decay times found in the literature for this type of device [63]. The steady-state dark current is ~3 × 10^−10^ A, which gives a photo-to-dark current ratio of ~10^6^, i.e., a value comparable with those found in the most photosensitive optoelectronic thin-film materials (e.g., a-Si:H, CdTe, CIGS, or CH_3_NH_3_PbI_3_ perovskites). These findings show that the ReSe_2_ multilayer crystals represent a solid choice for high-photosensitivity photodetectors.

## 5. Conclusions

A design for photodetector based on mechanically exfoliated ReSe_2_ crystal and GSC contacts in a lateral-heterostructure design was proposed. The device fabrication started with the growth of 200-μm-wide GSCs by CVD on Cu foil followed by transfer to a Si/SiO_2_ substrate. A ~100 nm-thick ReSe_2_ crystal was exfoliated and placed across two GSCs contacts by the “pick & place” method. This fabrication technique provides a simple and effective way to optically identify the desired atomically thin crystals amongst the high range of exfoliated flakes’ sizes and thicknesses while precisely controlling their orientation and position. Furthermore, since it is an all-dry procedure, it is possible to minimize the amount of additional contaminations. To compare the performance of GSC and Au contacts, a Au/ReSe_2_ photodetector was fabricated using the same crystal exfoliation and “pick & place” transfer methods. Owing to a low SBH with the ReSe_2_ crystal for the GSC (SBH ≈ 0.06 eV, as opposed to SBH ≈ 0.15 eV for the Au contacts), higher currents were measured in GSC-contacted devices in both the dark and illuminated which resulted in a higher gain and an enhanced linearity. The GSC/ReSe_2_ dark current was about 2×10^4^ higher than that of the Au/ReSe_2_ device. The GSC/ReSe_2_ photocurrent was one order of magnitude higher than similar ReSe_2_-based devices with metallic contacts reported in the literature. The responsivity, R_530nm_ = 4.7 × 10^2^ A/W and R_790nm_ = 3.3 × 10^2^ A/W was higher than in other reported devices. The external quantum efficiency under bias is in the expected range for this type of device with EQE_530nm_ = 1090% and EQE_790nm_ = 518%, confirming the non-blocking nature of the graphene contacts at V_bias_ = 2 V. Lastly, by implementing a CVD hBN passivation layer, an hBN/GSC/ReSe_2_ photodetector was fabricated. A ReSe_2_ crystal with a greater thickness was chosen to (i) maximize photon harvesting and (ii) study recombination effects of ReSe_2_. Under white light illumination, the hBN/GSC/ReSe_2_ device’s electrical response was characterized by a rise time of 0.5 s, coherent with the on/off cycles. The electrical conductivity as a function of the optical power saturated at ~10^3^ W/m, with a photo-to-dark current ratio of ~10^6^, a value that proofs ReSe_2_’s adequacy as an absorbing layer in photodetectors.

## Figures and Tables

**Table 1:** Photodetector characteristics tested under white light illumination.

	Au/ReSe_2_	GSC/ReSe_2_	hBN/GSC/ReSe_2_
I_ph_ (A)	4.5 × 10^−10^	1.5 × 10^−6^	4.6 × 10^−4^
A (m^2^)	4.9 × 10^−13^	5.1 × 10^−13^	5.2 × 10^−11^
F (V/m)	6.7 × 10^5^	3.2 × 10^5^	3.6× 10^5^
σ_ph_ (S/m)	1.4 × 10^−3^	9.2	24.7

**Table 2:** Comparison of key parameters from our ReSe_2_+Gr device and other reported ReSe_2_ based photodetectors.

Ref.	Photodetector	Thickness (nm)	Active Area, S (µm^2^)	Incident λ (nm)	Incident Power	I_ph_	R (A/W)	EQE (%)
Our work	Exfoliated ReSe_2_ and Au electrodes on Si/SiO_2_	58	26	530	3.3/2.9 mW cm^−2^	4.0/1.6 × 10^−11^ (V_bias_ = 2 V)	4.7 × 10^−2^	11
				790			2.1 × 10^−2^	3
Our work	Exfoliated ReSe_2_ and Gr electrodes on Si/SiO_2_	95	34	530	2.7/1.9 mW cm^−2^	4 × 10^−7^/2.1 × 10^−7^ A (V_bias_ = 2 V)	4.7 × 10^2^	1099
				790			3.3 × 10^2^	518
[67]	ML Gr/ReSe_2_/ML Gr heterostructure on Si/SiO_2_	14.5	18	220	0.14 mW cm^−2^	4.5 × 10^−5^ A (V_bias_ = 5 V)	1.2 × 10^6^	64
[38]	CVD ReSe_2_ and Cr/Au electrodes on Si/SiO_2_	4.2	5.8	808	5.7 × 10^2^ mW cm^−2^	9.7 × 10^−8^ A (V_bias_ = 5 V)	2.98	458
[65]	CVD ReSe_2_ and Cr/Au electrodes on Si/SiO_2_	0.71	19	850	6.1/7.0 mW cm^−2^	1 × 10^−8^ A/4 × 10^−9^ A (V_bias_ = 1 V)	8.4	12
				940			5.1	7
[12]	Exfoliated ReSe_2_ and Cr/Au electrodes on Si/SiO_2_	0.66	4	633	1 × 10^2^ mW cm^−2^	7.91 × 10^−8^ A (V_bias_ = 0.5 V)	17.8	3048
[66]	Exfoliated ReSe_2_ and Cr/Au electrodes on Si/SiO_2_	65	N/A	633	2.48 mW cm^−2^	1 × 10^−8^ A (V_bias_ = 1 V)	2.22	4
[68]	Exfoliated ReSe_2_ and Ti/Pd electrodes on Si/SiO_2_	80	25	785	1 mW cm^−2^	5.9 × 10^−8^ A (V_bias_ = 5 V)	4.3 × 10^3^	6791
[44]	Exfoliated ReSe_2_ and Ti electrodes on Si/SiO_2_	50.4	25	405	1 nW	3.62 × 10^−7^ A μm^−1^ (V_bias_ = 5 V)	1.1 × 10^3^	3367
[68]	Exfoliated ReSe_2_ and Pt electrodes on Si/SiO_2_	35	25	520	10 nW	1.61 × 10^−7^ A μm^−1^ (V_bias_ = 5 V)	79.99	191
[44]	Exfoliated ReSe_2_ + Mose_2_ heterostructure and Cr/Au electrodes on Si/SiO_2_	60	141	633	5.15 mW cm^−2^	4.9 × 10^−8^ A (V_bias_ = 1 V)	6.75	1266
[62]	Exfoliated ReSe_2_ p-doped with HCl and Pt electrodes on Si/SiO_2_	35	25	520	10 nW	6.32 × 10^−7^ A μm^−1^ (V_bias_ = 5 V)	3.144 × 10^2^	750
[69]	Exfoliated Mo:ReSe_2_ and Cr/Au electrodes on Si/SiO_2_	4.5	336	633	2 × 10^1^ mW cm^−2^	2.2 × 10^−6^ A (V_bias_ = 1 V)	55.5	109
[70]	Exfoliated ReSSe and Ti/Au electrodes on Si/SiO_2_	3	22	532	3.2 mW cm^−2^	5 × 10^−9^ A μm^−1^ (V_bias_ = 5 V)	8	19

## Data Availability

Not applicable.
